# Thermal Insulation and Compressive Strength of Lightweight Geopolymer Foam Concrete Exposed to Accelerated Weathering by Carbonation, Salt Fog and UV Light

**DOI:** 10.3390/ma19010012

**Published:** 2025-12-19

**Authors:** Gabriela A. de la Rosa-Corral, Ramón Corral-Higuera, Susana P. Arredondo-Rea, Andrés Castro-Beltrán, Anabel De la Cruz-Delgado, Alfredo Martinez-Garcia, Víctor M. Orozco-Carmona

**Affiliations:** 1 Departamento de Metalurgia e Integridad Estructural, Centro de Investigación en Materiales Avanzados, Avenida Miguel de Cervantes Saavedra 120, Chihuahua C.P. 31136, Mexico; gabriela.delarosa@cimav.edu.mx (G.A.d.l.R.-C.); anabel.delacruz@cimav.edu.mx (A.D.l.C.-D.); 2 Facultad de Ingeniería Mochis, Universidad Autónoma de Sinaloa, Fuente de Poseidón y Ángel Flores s/n, Ciudad Universitaria, Los Mochis C.P. 81223, Mexico; ramon.corral@uas.edu.mx (R.C.-H.); paola.arredondo@uas.edu.mx (S.P.A.-R.); andres.castro@uas.edu.mx (A.C.-B.)

**Keywords:** geopolymeric foam concrete, thermal conductivity, compressibility strength, accelerated weathering

## Abstract

This study investigates the deterioration of the thermal and mechanical properties of geopolymer foam concrete (GFC) subjected to accelerated weathering through carbonation, salt fog, and UV radiation. GFC blocks were synthesized using metakaolin as the aluminosilicate precursor, activated with an alkaline solution consisting of 8 M NaOH and sodium silicate (Na_2_SiO_3_) at a NaOH/Na_2_SiO_3_ ratio of 0.51 wt.%. A 30% (*v*/*v*) H_2_O_2_ solution served as the foaming agent, and olive oil was used as the surfactant. Accelerated carbonation tests were conducted at 25 ± 3 °C and 40 ± 3 °C, under 60 ± 5% relative humidity and 5% CO_2_, with carbonation depth, carbonation percentage, density, porosity, and thermal conductivity evaluated over a 7-day period. In parallel, specimens were exposed to salt fog and UV radiation for 12 weeks in accordance with ASTM B117-19 and ASTM G154-23, respectively. Compressive strength was monitored every week throughout the exposure period. Results show that carbonation temperature governs the type and kinetics of carbonate formation. The carbonation process, at 40 °C for 7 days, increased the density and reduced the porosity of GFC, resulting in a ~48% increase in thermal conductivity. Salt fog exposure led to severe mechanical degradation, with NaCl penetration reducing compressive strength by 69%. In contrast, UV radiation caused only minor deterioration, decreasing compressive strength by up to 7%, likely due to surface-level carbonation.

## 1. Introduction

The concept of sustainability in buildings is influenced by a range of factors, including environmental, economic, social, ecological, technical, and technological considerations [1,2,3,4]. Green and sustainable buildings help mitigate adverse impacts on the environment, economy, and society [5,6,7]. Consequently, current trends in the housing and construction industry are driving the development of sustainable building materials [8,9,10,11]. Within green building design, energy efficiency is a key criterion, and thermal insulation materials play a critical role [1,12,13,14,15]. Cellular concrete is frequently used as a thermal insulation material in sustainable construction [16,17,18,19,20,21].

GFC is a recent innovation that combines the benefits of foam concrete and geopolymer technology [9,22,23,24,25]. GFC is a lightweight material composed of an alkali-activated geopolymer matrix and a foaming agent that together generate a cellular microstructure [26]. Geopolymers are inorganic polymers formed by the alkali activation of aluminosilicate precursors—materials rich in silica and alumina [27,28,29,30,31,32]. Metakaolin (MK), derived from the calcination of natural kaolin clay at approximately 750 °C, is the most commonly used aluminosilicate precursor in geopolymer concrete production [33].

Recent studies have focused on the durability of GFC, as this is a key factor limiting the broader application of geopolymer technology in the construction sector. Enhancing the durability of GFC could significantly expand its use as a thermal insulation material in construction [34]. In terms of atmospheric degradation, carbonation is a critical durability concern for concrete structures. In ordinary Portland cement (OPC) concrete, carbonation occurs when atmospheric CO_2_ penetrates the matrix and reacts with calcium hydroxide (Ca(OH)_2_) to form calcium carbonate (CaCO_3_). This reaction lowers the pH of the concrete, which can initiate and accelerate the corrosion of embedded steel reinforcement [35]. In contrast, carbonation in geopolymer concrete primarily involves the reaction of NaOH with CO_2_ to form sodium carbonate hydrates [36]. Badar et al. [37] identified the formation of nahcolite (NaHCO_3_) and natron (Na_2_CO_3_·10H_2_O) in low-calcium fly ash geopolymers cured at 80 °C for 72 h [38]. Studies have shown that high-density geopolymers exhibit greater resistance to carbonation, whereas low-density formulations (with higher porosity and interconnected pore structures) facilitate CO_2_ diffusion and thus accelerate carbonation [6,39]. Interestingly, some research reports that carbonation can increase the density and compressive strength of certain geopolymer systems [39].

While the resistance of GFC to carbonation and its impact on mechanical properties have been relatively well studied, there remains insufficient data on how accelerated carbonation affects its thermal insulation performance. Moreover, no studies to date have reported on the compressive strength of GFC subjected to combined accelerated weathering from salt fog and UV radiation. Therefore, this study investigates the evolution of thermal and mechanical properties in GFC exposed to accelerated weathering conditions, specifically carbonation (at 25 °C and 40 °C), salt fog, and UV light.

## 2. Materials and Methods

### 2.1. Preparation of Starting Materials

Metakaolin (MK) (Metastar 501, Watson Phillips, Naucalpan de Juárez, Mexico) was used as the aluminosilicate precursor. Its physical properties include a density of 2.5 g/cm^3^, an average particle size of 1.6 µm, and a BET surface area of 3.87 m^2^/g. The chemical composition of the MK was determined by X-ray fluorescence spectroscopy (XRF) and is summarized in Table 1.

The alkaline activator was prepared by mixing an 8 M NaOH solution (Macron, Mexico) with sodium silicate (Na_2_SiO_3_, 10.6 wt.% Na_2_O and 26.5 wt.% SiO_2_; Sigma-Aldrich, St. Louis, MO, USA) at a NaOH/Na_2_SiO_3_ ratio of 0.51 wt.%. A 30% (*v*/*v*) H_2_O_2_ solution (J.T. Baker, Radnor, PA, USA) was used as the foaming agent, and extra virgin olive oil (Ragasa Industrias, Monterrey, Mexico) served as the surfactant.

### 2.2. Paste Preparation for GFC

The activating solution and metakaolin (MK) were mixed at a solid-to-liquid ratio of 1.14. The mixture was poured into a laboratory mixer (Hobar, Troy, OH, USA) and vigorously mixed for two minutes. Subsequently, 3 wt.% of the foaming agent (30% H_2_O_2_ solution) was added and homogenized for an additional two minutes [40]. Finally, 1.5 wt.% of surfactant (extra virgin olive oil) was incorporated and mixed for one more minute. The fresh paste was then cast into cylindrical molds. Carbonation accelerated process at 40 °C for 7 days, induced increases in density and reductions in porosity, and increased thermal conductivity of GFC by about 48%. The samples were pre-cured at ambient temperature for 24 h, followed by curing at 60 °C for another 24 h.

### 2.3. Weathering Tests on GFC Blocks

#### 2.3.1. Accelerated Weathering Due to Carbonation

The GFC blocks were exposed to an accelerated carbonation chamber designed and constructed at the Advanced Materials Research Center (Chihuahua, Mexico). The tests were conducted at two temperatures: 25 ± 3 °C and 40 ± 3 °C, under 60 ± 5% relative humidity and a CO_2_ concentration of 5%. The temperatures of 25 °C and 40 °C were selected based on the typical diurnal temperature ranges commonly observed in ambient environments. Carbonation progression was monitored daily over a 7-day period.

Carbonation depth was evaluated using phenolphthalein (Sigma-Aldrich, St. Louis, MO, USA) as a pH indicator. Phenolphthalein changes color from fuchsia (at pH ≥ 10) to colorless (at pH ≤ 8.3), with intermediate pink shades appearing between pH 10 and 8.3 [37]. Digital images of the cross-sections were analyzed using ImageJ software (version 1.54d) to calculate the percentage of carbonated surface based on color contrast. Additionally, the density, porosity, and thermal conductivity of the GFC samples were measured.

#### 2.3.2. Accelerated Weathering Due to Salt Fog and UV Light

The GFC samples were subjected to salt fog and UV radiation exposure. Salt fog testing was performed in a Q-Fog chamber (Q-Lab, Westlake, OH, USA) according to ASTM B117-19 [41], while UV exposure was carried out in a Q-UV chamber (Q-Lab, Westlake, OH, USA) following ASTM G154-23 [42]. Both exposures lasted 12 weeks. Compressive strength was measured weekly using a universal testing machine (MTS, Eden Prairie, MN, USA) to track the chronological deterioration induced by these environmental stressors.

### 2.4. Characterization Techniques

#### 2.4.1. Density and Porosity

The apparent density (*ρ_b_*) was determined using Archimedes’ principle in accordance with ASTM C188-25, using Equation (1) [43].

(1) $$\rho_{b}=\frac{m_{1}}{m_{1}-m_{2}}\rho_{w}$$

where *m*_1_ is weight of the dry material, m_2_ is the weight submerged in water, and *ρ_w_* is the density of the liquid. True density *ρ_τ_* (g/cm^3^) was measured using an Ultrapycnometer 1000 (Quantachrome Instruments, Boynton Beach, FL, USA) with nitrogen gas displacement (Praxair, Danbury, CT, USA). Total porosity (vol.%) was then calculated from apparent and true densities using Equation (2).

(2) $$Total\;porosity=1-\frac{\rho_{b}}{\rho \tau }*100$$

#### 2.4.2. Thermal Conductivity Measurement

Thermal conductivity (*λ*) was measured using the guarded heat flow meter method, following ASTM E1530-25 [44]. Tests were conducted with a conductometer model 2020 (Unitherm, Ciudad López Mateos, Mexico) apparatus on cylindrical pellets (5.08 cm in diameter and 2.54 cm in height).

#### 2.4.3. Powders X-Ray Diffraction

XRD patterns were acquired using a D8 Advance diffractometer (Bruker, Mannheim, Germany) with Cu Kα radiation, scanning from 5° to 60° 2θ, with a step size of 0.016° and a counting time of 4 s per step. Phase identification and semi-quantitative analysis were performed using X’Pert HighScore Plus software (version 2.2b).

#### 2.4.4. Fourier Transform-Infrared Spectroscopy

FTIR spectra were collected on an Affinity-1S spectrophotometer (Shimadzu, Kyoto, Japan) over the wavenumber range of 4000–400 cm^−1^.

#### 2.4.5. Scanning Electron Microscopy

Microstructural analysis was performed using an SU3500 SEM (Hitachi, Kyoto, Japan) operated at 10 kV under high vacuum, equipped with energy-dispersive X-ray spectroscopy (EDS) for elemental mapping.

## 3. Results and Discussion

### 3.1. Effect of Temperature on Accelerated Weathering by Carbonation

#### 3.1.1. Carbonation Depth

**Figure 1:**
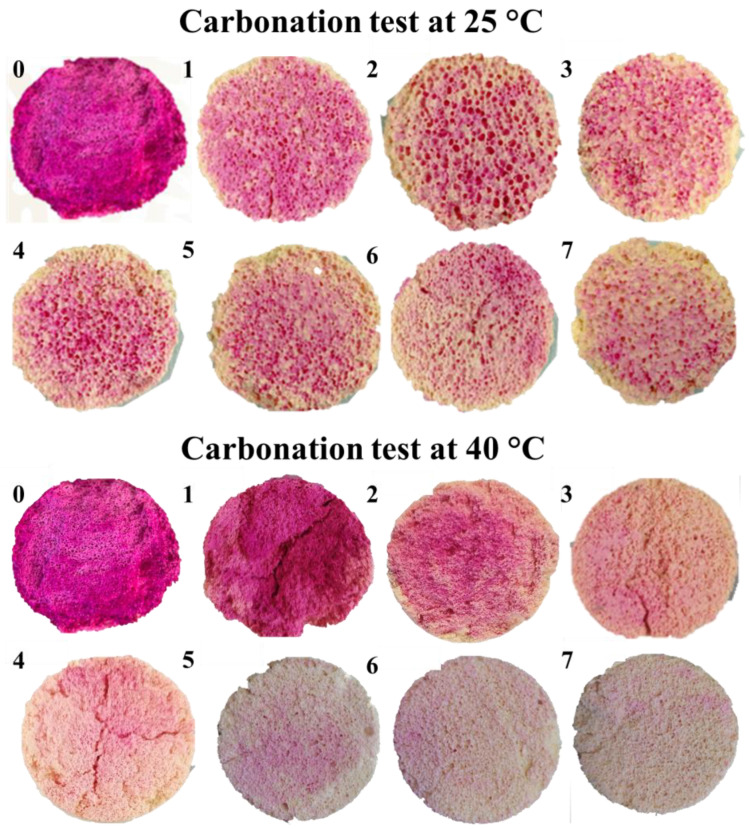
Carbonation gradient in GFC samples exposed at 25 °C and 40 °C over seven days, visualized using phenolphthalein indicator.

Figure 1 shows the carbonation gradient in GFC samples exposed at 25 °C and 40 °C over seven days. Discoloration of the phenolphthalein indicator reflects the decrease in pH due to carbonation. At 25 °C, the color change progresses gradually over time but does not fully discolor. Meanwhile, at 40 °C, the color change is slow during the first two days, and then discoloration occurs faster, indicating faster carbonation kinetics.

**Figure 2:**
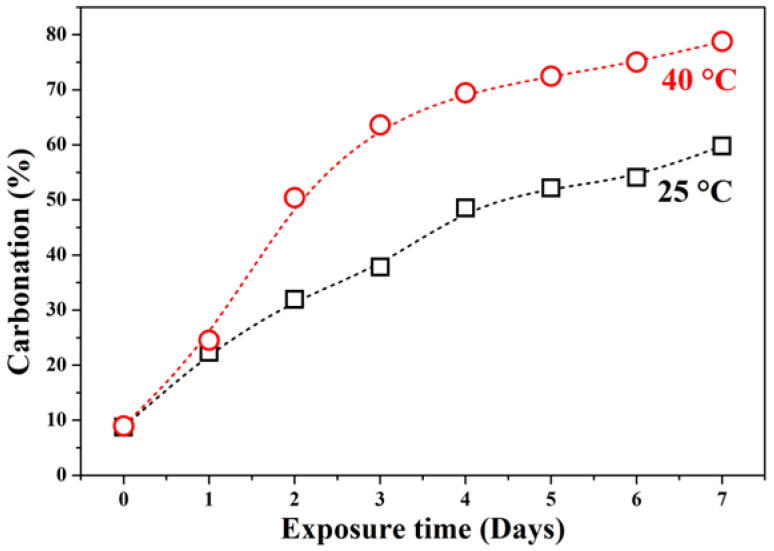
Quantitative analysis of carbonation depth in GFC exposed at 25 °C and 40 °C, based on image analysis of pH indicator discoloration.

Figure 2 presents the quantitative carbonation percentage derived from color contrast analysis. At 25 °C, carbonation increases sharply during the first five days and then plateaus. In contrast, at 40 °C, carbonation rises rapidly during the first four days, reaching a maximum of ~80% surface carbonation by day 7. The accelerated carbonation at higher temperatures is attributed to enhanced CO_2_ solubility in pore water and increased reaction kinetics.

The initial rapid carbonation (days 0–4) at both temperatures likely results from readily available reactive sites (e.g., NaOH) near the surface. The subsequent slowdown may be due to pore blockage by sodium carbonate products (e.g., natrite, trona), which hinders further CO_2_ diffusion into the matrix. The highly porous and interconnected structure of GFC facilitates CO_2_ ingress, but temperature critically influences both the rate and nature of carbonation products formed.

#### 3.1.2. Density and Porosity Analysis

**Figure 3:**
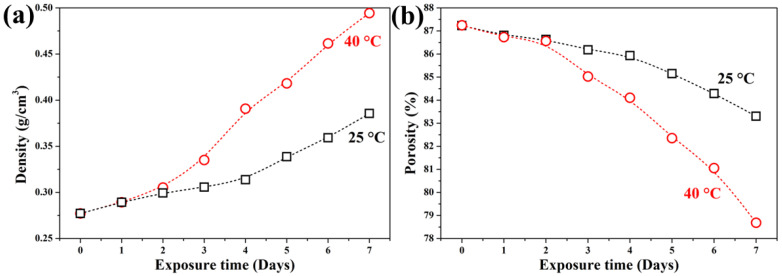
Evolution of (**a**) density and (**b**) porosity in GFC samples exposed to accelerated carbonation at 25 °C and 40 °C over 7 days.

Figure 3 illustrates the evolution of bulk density and porosity during carbonation. The uncarbonated GFC exhibited a bulk density of 0.277 g/cm^3^ and a porosity of 87.3%, consistent with values reported in the literature for chemical GFC [45]. After 7 days of carbonation at 25 °C, the samples reached a density of 0.373 g/cm^3^ and a porosity of 83.30%. At 40 °C, the corresponding values were 0.494 g/cm^3^ and 78.68%, respectively.

No significant changes in density or porosity were observed during the first two days of exposure. However, from day 3 onward, both properties shifted markedly. After 7 days at 25 °C, density increased by 35% and porosity decreased by 4.5%. At 40 °C, the changes were more pronounced: density rose by 78%, and porosity dropped by 10%. These results confirm that elevated temperature accelerates the formation and deposition of dense carbonate phases within the pore structure, thereby reducing porosity and increasing bulk density.

The pore-filling effect explains the deceleration in carbonation rate after day 4 (Figure 2); as carbonation products progressively seal the surface and limit CO_2_ access to deeper regions.

#### 3.1.3. Physicochemical Analysis

**Figure 4:**
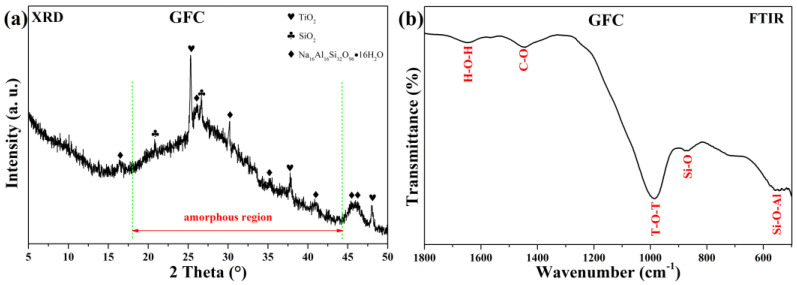
(**a**) XRD pattern and (**b**) FTIR spectrum of uncarbonated GFC.

Figure 4 shows the XRD pattern and FTIR spectrum of uncarbonated GFC cured at 60 °C for 24 h. The XRD diffractogram (Figure 4a) displays a broad hump between 17° and 45° 2θ, characteristic of the amorphous geopolymer gel formed during alkali activation [46,47,48]. Crystalline phases identified include anatase (TiO_2_, AMCSD-0010735), quartz (SiO_2_, AMCSD-0000789), and analcime (Na_16_Al_16_Si_32_O_96_·16H_2_O, AMCSD-0000654).

The FTIR spectrum (Figure 4b) shows characteristic bands at 1645 cm^−1^ (O–H bending), 1004 cm^−1^ (asymmetric T–O–T stretching, T = Si/Al), 879–867 cm^−1^ (Si–O bending), and 561–540 cm^−1^ (Si–O–Al bending) [49,50,51]. The broadband near 1100 cm^−1^ arises from external tetrahedral linkages, while the weak band at 1440 cm^−1^ suggests the presence of atmospheric or structurally bound carbonates [52].

**Figure 5:**
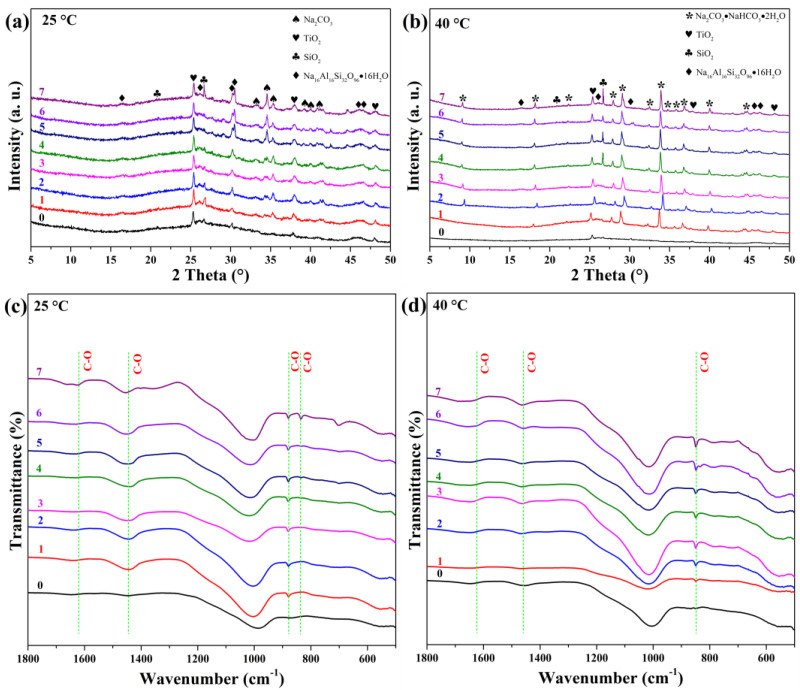
XRD patterns and FTIR spectra of GFC exposed to accelerated carbonation at (**a**,**c**) 25 °C and (**b**,**d**) 40 °C for up to 7 days.

Figure 5 illustrates the structural evolution during carbonation. At 25 °C (Figure 5a,c), XRD reveals the emergence of natrite (Na_2_CO_3_, AMCSD-0005044) from day 1, with increasing crystallinity over time. FTIR shows growing intensities at 1620, 1440, and 879 cm^−1^ (assigned to C–O vibrations), confirming carbonate formation. The appearance of a band at 836 cm^−1^ on day 7 indicates hydrated carbonates. Saturation is suggested by the plateau in band intensity after day 5.

At 40 °C (Figure 5b,d), XRD shows rapid formation of trona (Na_2_CO_3_·NaHCO_3_·2H_2_O, AMCSD-0009779) within the first day. Peak shifts during the first two days may reflect non-stoichiometric or hydrated phase transitions. FTIR confirms hydrated carbonates via bands at 1620, 1440, and 836 cm^−1^.

These results demonstrate that carbonation temperature dictates the type of carbonate phase: anhydrous natrite dominates at 25 °C, while hydrated trona forms at 40 °C, consistent with literature reporting temperature-dependent carbonate speciation [52,53].

**Figure 6:**
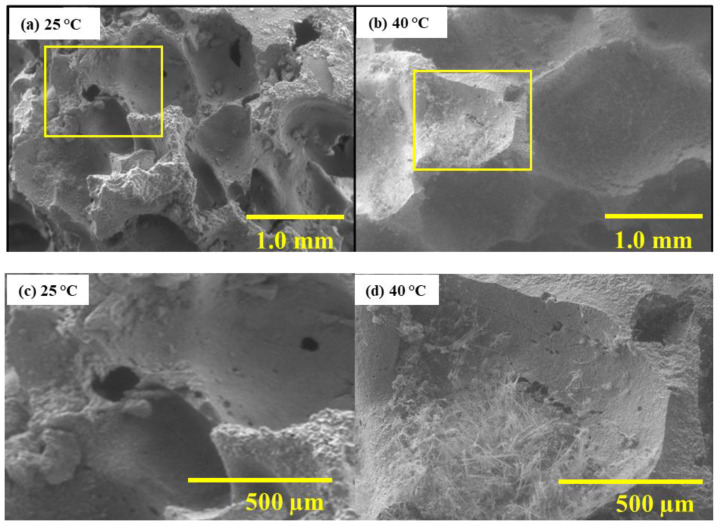
SEM micrographs of GFC surface after carbonation at 25 °C and 40 °C.

Figure 6 shows SEM micrographs of cross-sections. At 25 °C, no internal carbonation products are visible, suggesting surface pore clogging by natrite crystals limits CO_2_ penetration. In contrast, at 40 °C, fibrous trona crystals grow within the pore network, indicating deeper CO_2_ diffusion and internal crystallization.

#### 3.1.4. Impact of Carbonation Temperature on Thermal Insulation Properties

**Figure 7:**
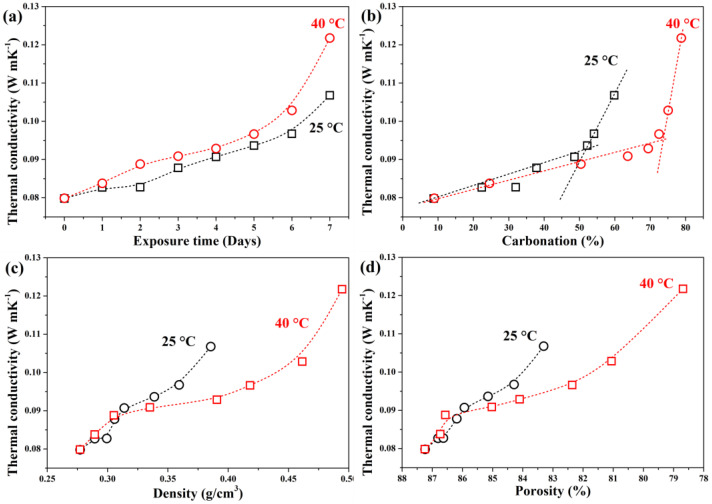
Effect of (**a**) exposure time, (**b**) carbonation percentage, (**c**) density, and (**d**) porosity on thermal conductivity of GFC carbonated at 25 °C and 40 °C, respectively.

Figure 7 shows the influence of carbonation on thermal conductivity. Uncarbonated GFC exhibited a thermal conductivity of 0.083 W/m·K, comparable to literature values for low-density GFC (~0.088 W/m·K at 0.28 g/cm^3^) [45]. The low baseline conductivity stems from high porosity and limited solid conduction pathways; trapped air and bound water further reduce heat transfer [54,55,56].

After 7 days, thermal conductivity increased to 0.107 W/m·K (25 °C) and 0.1217 W/m·K (40 °C), respectively. The higher value at 40 °C correlates with the formation of hydrated carbonates (confirmed by XRD/FTIR), as water molecules enhance thermal conduction [55].

Initially (days 0–5), thermal conductivity rose similarly at both temperatures (~15% increase), reaching ~0.094–0.096 W/m·K; see Figure 7a). However, during days 5–7, the increase accelerated at 40 °C (0.122 W/m·K, about 48% total rise) but remained linear at 25 °C (0.107 W/m·K, about 28% total rise). This divergence aligns with the exponential increase in density and pore filling observed at higher temperatures (Figure 7c,d).

Thermal conductivity showed a linear relationship with density/porosity at 25 °C but an exponential trend at 40 °C after day 2, highlighting the critical role of temperature in altering heat transfer mechanisms via microstructural evolution. Therefore, the increase in temperature accelerates the carbonation process, reducing the insulation properties of the GFC.

### 3.2. Accelerated Weathering by Salt Fog and UV Light

#### 3.2.1. Physicochemical Analysis

**Figure 8:**
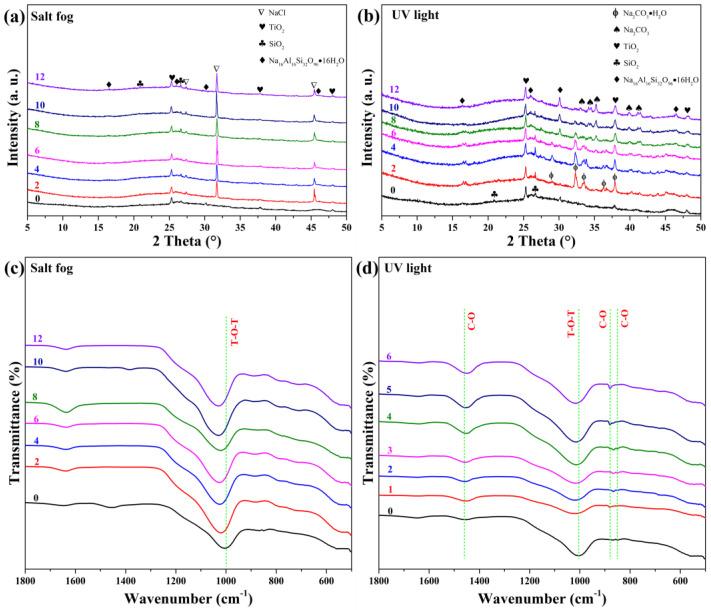
XRD patterns and FTIR spectra of GFC after (**a**,**c**) salt fog and (**b**,**d**) UV exposure, respectively.

Figure 8 presents XRD and FTIR results after salt fog and UV exposure. Salt fog exposure (Figure 8a,c) introduced halite (NaCl, AMCSD-0000641) peaks from week 1, confirming NaCl crystallization within the pores. FTIR shows increased intensity at 1645 cm^−1^ (O–H) and a shift of the T–O–T band from 1004 to 1025 cm^−1^, indicating solvation of aluminosilicate networks due to NaCl/moisture adsorption [57].

UV exposure (Figure 8b,d) induced formation of thermonatrite (Na_2_CO_3_·H_2_O, AMCSD-0009532) within two weeks, which gradually dehydrated to natrite (Na_2_CO_3_). The amorphous content increased slightly, and FTIR showed enhanced carbonate bands (1440, 880, and 840 cm^−1^), confirming UV-promoted carbonation, likely due to photochemical reactions or surface drying that favors CO_2_ uptake.

#### 3.2.2. Impact of Salt Fog and UV Light on GFC Compressive Strength Properties

**Figure 9:**
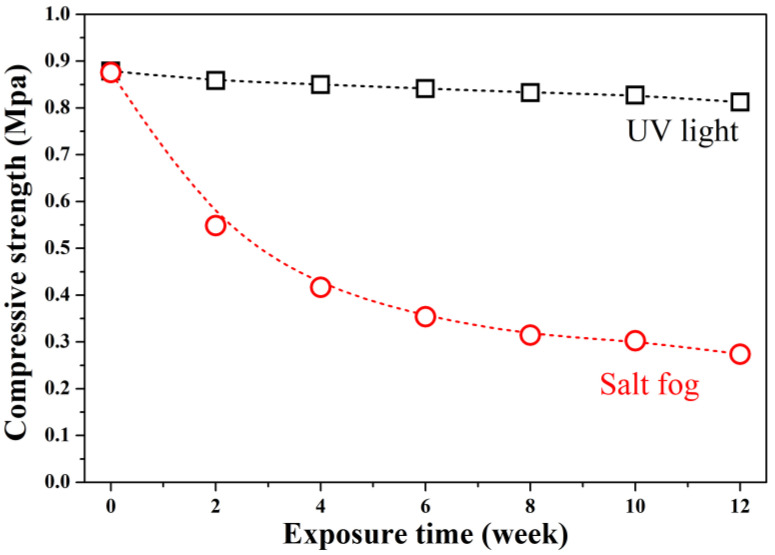
Compressive strength of GFC exposed to salt fog and UV radiation over 12 weeks.

Figure 9 shows compressive strength evolution. Uncarbonated GFC had a strength of 0.878 MPa, typical for ultra-lightweight foams (<1 MPa at ~0.28 g/cm^3^) [1]. After 12 weeks:

UV exposure caused a 7% strength loss (to 0.817 MPa), consistent with minor surface degradation.

Salt fog exposure led to severe deterioration; after 12 weeks of exposure, the compressive strength of the material decreased exponentially to 0.274 MPa, a reduction of 69% compared to its initial value.

The drastic loss under salt fog is attributed to NaCl crystallization pressure and chemical disruption of Al–O–Al and Si–O–Si bonds via ion exchange and hydration. In contrast, UV-induced carbonation had negligible mechanical impact.

## 4. Conclusions

This study investigated the effects of three accelerated weathering agents (carbonation at 25 °C and 40 °C, salt fog, and UV radiation) on the thermal and mechanical performance of metakaolin-based GFC.

Carbonation temperature critically controls the type and kinetics of carbonate formation: hydrated trona dominates at 40 °C, while anhydrous natrite forms at 25 °C. Higher temperature accelerates CO_2_ diffusion and pore-filling, increasing density by up to 78% and reducing porosity by 10% after 7 days.

The thermal conductivity of GFC increased by up to 48% (from 0.083 to 0.122 W/m·K) due to pore infilling and the presence of water in hydrated carbonates. The relationship between conductivity and density shifted from linear (25 °C) to exponential (40 °C), highlighting temperature-dependent microstructural effects.

Salt fog exposure caused severe mechanical degradation (69% strength loss), primarily due to NaCl penetration, crystallization pressure, and disruption of the geopolymer network via solvation of key functional groups.

UV radiation promoted mild surface carbonation but only reduced compressive strength by 7%, indicating minimal structural impact.

These findings underscore that while carbonation enhances thermal performance (via densification), it must be balanced against potential long-term durability risks, especially in marine environments where salt fog poses a severe threat to mechanical integrity. UV exposure, in contrast, is relatively benign for GFC applications.

## Figures and Tables

**Table 1:** Percentage of phases present in MK.

Phase	SiO_2_	Al_2_O_3_	Fe_2_O_3_	CaO	MgO	TiO_2_
wt. %	53.9	41.9	1.1	1.1	0.2	1.8

The phase composition was obtained by refining the XRD pattern of the MK.

## Data Availability

The original contributions presented in this study are included in the article. Further inquiries can be directed to the corresponding authors.
